# Cost-effectiveness of pembrolizumab plus chemotherapy vs. chemotherapy as first-line treatment for advanced biliary tract cancer in China and the US

**DOI:** 10.3389/fphar.2024.1393559

**Published:** 2024-08-14

**Authors:** Xianmei Luo, Tingting Cai, Jinyan Wu, Xingyu Li, Xiaofan Wang, Haiying Ma

**Affiliations:** ^1^ Department of Pharmacy, The Fourth Affiliated Hospital of China Medical University, Shenyang, China; ^2^ School of Pharmacy, China Medical University, Shenyang, China; ^3^ Key Laboratory of Birth Defects and Related Diseases of Women and Children of MOE, State Key Laboratory of Biotherapy, West China Second University Hospital, Sichuan University, Chengdu, China

**Keywords:** pembrolizumab, advanced biliary tract cancer, Markov model, cost-effectiveness, China, US

## Abstract

**Background:** Pembrolizumab is a potentially valuable treatment. However, patients, doctors, and healthcare decision-makers are uncertain about its cost-effectiveness and an appropriate pricing for this new therapy. This study aims to appraise the cost-effectiveness of pembrolizumab as a first-line treatment for advanced biliary tract cancer (BTC) patients in China and the United States (US).

**Methods:** A Markov model was constructed from the perspectives of healthcare systems in both China and the US for pharmacoeconomic evaluation. Patient baseline characteristics and key clinical data were sourced from the KEYNOTE-966 trial (ClinicalTrials.gov, NCT04003636). Costs and utilities were collected from drug cost websites and published literature. Cumulative costs (in USD), life years (LYs), quality-adjusted life years (QALYs), and incremental cost-effectiveness ratios (ICERs) were measured and compared. Price simulations were conducted under given willingness-to-pay (WTP) thresholds to provide pricing scheme references. The model’s robustness was analyzed through one-way sensitivity analysis and probabilistic sensitivity analysis.

**Results:** Basic data analysis illustrates that pembrolizumab ($2662.41/100 mg) in combination with chemotherapy regimen was not cost-effective relative to chemotherapy regimens at the WTP threshold of $38,201.19 in China, and the additional cost relative to chemotherapy regimens was $77,114.94 (ICER $556,689.47/QALY) while increasing 0.14 QALYs. Pembrolizumab ($54.71/1 mg) also increased efficacy by 0.14 QALYs in the US, but remained also not cost-effective at the US WTP threshold of $229,044, and the total cost increased by $160,425.24 (ICER $1,109,462.92/QALY).

**Conclusion:** Compared with chemotherapy, pembrolizumab plus chemotherapy reduces the disease of burden. However, at its current price, it may not be a cost-effective treatment for advanced BTC in both China and the US. This study can aid decision-makers in making optimal choices.

## Introduction

Biliary tract cancer (BTC) comprises a group of complex epithelial malignant tumors originating from the intrahepatic or extrahepatic bile ducts and the gallbladder. Risk factors for this disease include primary sclerosing cholangitis, Caroli’s disease, intrahepatic bile duct stones, and liver fluke infection (Clements et al., 2020; Valle et al., 2021). BTC accounts for approximately 3% of all gastrointestinal tumors, ranking as the sixth most common digestive system tumor after colorectal cancer, pancreatic cancer, liver cancer, gastric cancer, and esophageal cancer (Fitzmaurice et al., 2015; Khan et al., 2019). In the early stages, BTC is often asymptomatic, leading to delayed diagnosis, up to 80% of patients are diagnosed as unresectable or metastatic by the time of confirmation (Peirce et al., 2023). The 5-year relative survival rate for patients with BTC at any stage is 9%–11%, with only a 2% survival rate for metastatic BTC, earning it the title of the “Hidden Cancer King” (Tam et al., 2022; American Cancer Society, 2023). Age-adjusted incidence rates for BTC are about one-third higher in males than in females, with lower incidence rates in high-income countries (2 cases per 100,000 people annually). However, in epidemic regions of China, the incidence rate is 40 times higher than that in high-income countries (Everhart and Ruhl, 2009; Valle et al., 2021). Analysis of the 2019 SEER database registration data in the US manifests a rising incidence and mortality rate over time for intrahepatic bile duct cancer, while the incidence and mortality rates for extrahepatic bile duct cancer and gallbladder cancer remain relatively stable (Jiang et al., 2022).

In the evolving systemic regimen of BTC, neoadjuvant therapy is attractive because this treatment strategy has the potential to improve local and distant control, achieve R0 resection and prevent distant metastases (Rizzo and Brandi, 2021a). A meta-analysis conducted by Horgan et al. showed that adjuvant chemotherapy and chemoradiotherapy were beneficial for patients with BTC, while no benefit was observed with radiotherapy alone (Horgan et al., 2012; Rizzo and Brandi, 2021b). Shimoyama et al. (2023)’s research demonstrates that the prognosis of combination therapy is significantly superior to that of monotherapy. From 2010 to 2021, the systematic combination therapy of gemcitabine and cisplatin has been the standard treatment for biliary tract cancer. However, the rapid development of resistance has become a bottleneck for chemotherapy drugs like cisplatin, and the emergence of multidrug resistance is particularly concerning, as over 90% of deaths in cancer patients undergoing treatment with traditional or novel chemotherapy drugs are caused by multidrug resistance (Bukowski et al., 2020). Therefore, alternative treatment strategies need to be explored. The latest treatment method for BTC involves immune checkpoint inhibitors (ICIs), comprising programmed cell death receptor-1 (PD-1) and its ligand (PD-L1) inhibitors. Eastern Cooperative Oncology Group (ECOG) performance status score of 0 or 1 has been shown to reduce the risk of death or progression in this population, either alone or in combination, in patients on immunotherapy (Mollica et al., 2023). In 2022, the first immunotherapy for BTC, the PD-L1 inhibitor durvalumab, was approved for inclusion in the combination with gemcitabine and cisplatin, as the TOPAZ-1 trial showed that durvalumab, in combination with gemcitabine and cisplatin, distinctly improved overall survival (OS) (Oh et al., 2022). The PD-1 inhibitor pembrolizumab, in Alshari et al. (2019)’s study, was used as a palliative treatment after progression in BTC and reported two cases of advanced BTC patients with complete tumor resolution after pembrolizumab treatment. Additionally, two independent studies of pembrolizumab, the KEYNOTE-028 trial (phase 1b) and the KEYNOTE-158 trial (phase 2), attested that pembrolizumab monotherapy provides durable antitumor activity regardless of PD-L1 expression and is associated with manageable toxicity in advanced BTC patients with no other standard treatment options, thus proving the effectiveness and safety of pembrolizumab (Piha-Paul et al., 2020). The global phase 3 study, KEYNOTE-966, as the first global study of a PD-1 inhibitor and the second to show a significant improvement in OS for patients with BTC, demonstrated a 1.8-month improvement in median OS and a 0.9-month improvement in median progression-free survival (PFS) compared with chemotherapy alone, further confirming the efficacy and safety of pembrolizumab in combination with chemotherapy (Kelley et al., 2023).

Before the KEYNOTE-966 trial, “Guidelines for the Diagnosis and Treatment of Malignant Biliary Tumors 2022”compiled by the Chinese Society of Clinical Oncology (CSCO), pembrolizumab is only indicated for patients with MSI-H/dMMR in first- and second-line treatment for BTC and for second-line category Ⅲ recommendation. In the “Diagnosis and Treatment Guidelines for Primary Biliary Tract Cancer (Version 2023.2)” published by the National Comprehensive Cancer Network (NCCN) in the US, pembrolizumab is recommended for patients with MSI-H/dMMR in first- and second-line therapy for BTC and for patients with TMB-H in second-line therapy. However, factors such as treatment duration, cost, and technology have limited its widespread application (Guidelines Working Committee of Chinese Society of Clinical Oncology, 2022; Zhao Y. et al., 2023; National Comprehensive Cancer Network, 2023). The success of the KEYNOTE-966 trial enables the use of pembrolizumab in first-line treatment for advanced BTC, expanding its application range. Given its confirmed effectiveness and safety, its economic viability should be considered, prompting a need for further research on pharmacoeconomic evaluation. Cost-utility analysis in pharmacoeconomics considers both economic costs and health outcomes, allowing healthcare decision-makers to choose the most cost-effective options. In the evaluation methods of pharmacoeconomics, complex chronic diseases often employ Markov models for simulation. Therefore, this study establishes a Markov model to assess the cost-utility of pembrolizumab in combination with chemotherapy for advanced BTC from the perspectives of the healthcare systems in China and the US. This aims to provide guidance for clinical physicians and healthcare decision-makers in optimizing the allocation of limited medical resources.

## Methods

### Materials and methods

This study utilized TreeAge Pro 2022 software to construct a Markov model, estimating the cost-effectiveness of two treatment strategies–chemotherapy alone and pembrolizumab plus chemotherapy. The research perspective encompassed the healthcare systems of China and the US, incorporating direct medical costs within the healthcare systems, and referring to the utility value in the published literature. Patient survival curves, administration methods, probabilities of severe adverse reactions (AEs), and subsequent treatment ratios were obtained from the KEYNOTE-966 trial results. Model results were expressed as total costs, LYs, QALYs, and ICERs. Discount rates for costs and utilities in China were set at 5%, while in the US, a 3% rate was applied (Sanders et al., 2016; Chinese Pharmaceutical Association, 2020). Following the World Health Organization’s recommendations, the willingness-to-pay (WTP) threshold was defined as three times the 2022 *per capita* GDP in China ($12,733.73), equating to $38,201.19, and three times the 2022 *per capita* GDP in the US ($76,348), amounting to $229,044 (National Bureau Of Statistics Of China, 2022).

### Model establishment

**FIGURE 1:**
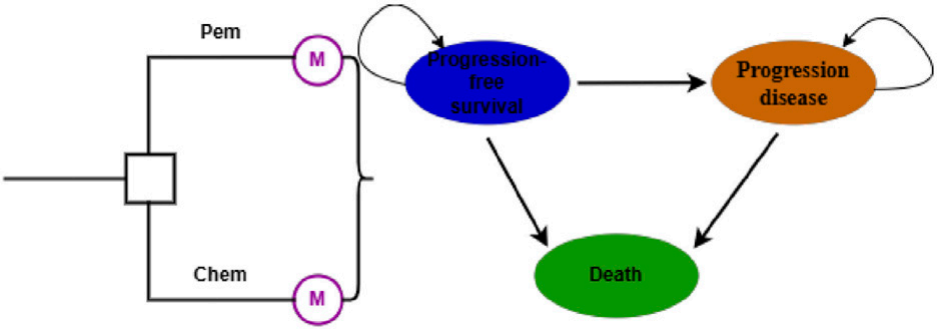
Markov model. Pem, pembrolizumab; Chem, chemotherapy.

The Markov model comprises three mutually exclusive health states: PFS, Progression Disease (PD), and Death (Figure 1). All patients start in the initial state of PFS, with the ultimate endpoint being death. The model cycle aligns with the dosing cycle (21 days). To more accurately simulate the transition process, the model undergoes half-cycle correction (Ye et al., 2023). Immunotherapy exhibits a delayed effect and may continue to confer benefits beyond the treatment period, thus long-term data are employed for analysis to avoid inaccuracies in results. The model’s operational period is set at 10 years to simulate the entire lifespan of the patients (Zhao Q. et al., 2023).

### Patients and intervention

The basic medical data used in this economic evaluation are derived from a large randomized, double-blind, placebo-controlled global phase 3 study (KEYNOTE-966). Participants meeting the study criteria were aged 18 or older, with untreated, unresectable, locally advanced or metastatic BTC. The only allowed systemic treatment prior to study participation was neoadjuvant or adjuvant therapy completed at least 6 months before the diagnosis of unresectable or metastatic disease. Measurable disease according to Response Evaluation Criteria in Solid Tumors version 1.1; ECOG performance status score of 0 or 1; concurrent hepatitis B patients initiated antiviral treatment at least 4 weeks before the start of the study therapy, with a viral load below 100 IU/mL.

Pembrolizumab (200 mg) or saline placebo is intravenously injected every 3 weeks. Gemcitabine (1,000 mg/m^2^) and cisplatin (25 mg/m^2^) are intravenously injected on days 1 and 8 of a 3-week cycle. Treatment continues until disease progression, unacceptable toxicity, investigator decision, withdrawal of consent, or other reasons, whichever comes first. Pembrolizumab and placebo are limited to 35 cycles, cisplatin is limited to 8 cycles, and there is no limit on the number of cycles for gemcitabine. Contrast-enhanced CT (preferred) or MRI of the chest, abdomen, and pelvis is performed every 6 weeks during the first 4 weeks before randomization, every 6 weeks after the first study treatment, until week 54, and then every 12 weeks thereafter (Kelley et al., 2023).

### Clinical data inputs

Due to the challenges of long-term tracking in clinical trials and limited follow-up time to assess the impact of drug administration on the target population, this study utilized GetData software (http://www.getdata-graph-digitizer.com/) to extract individual patient data from Kaplan-Meier (K-M) curves for PFS and OS in the KEYNOTE-966 trial. R software (version 4.2.2; https://www.r-project.org) was utilized for data reconstruction. Standard parametric models (exponential, Gompertz, Weibull, loglogistic, lognormal, gamma) were used to fit the reconstructed data, and visual inspection combined with goodness-of-fit tests [Akaike information criterion (AIC) and Bayesian information criterion (BIC)] selected the best-fitting distribution. The best-fitting distribution was then extrapolated, and the cumulative survival rate obtained was used to calculate the transfer probability (Diaby et al., 2014).

### Cost

The direct medical expenses include drug costs, routine follow-up, adverse event expenses and subsequent treatment costs for disease progression. To simplify the calculation, AEs with a grade of ≥3AE and an incidence of ≥9% were selected for calculation, assuming all AEs occurred in the first cycle. The total cost of an AE is calculated by multiplying the cost of an individual AE by the corresponding probability of occurrence of the AE, and then summing the cost of all AEs. Chinese costs were converted based on the 2022 exchange rate (1.0 USD = 6.73 CNY). The subsequent treatment regimen for disease progression is based on recommendations from the CSCO “Guidelines for Diagnosis and Treatment of Malignant Biliary Tumors 2022” (Guidelines Working Committee of Chinese Society of Clinical Oncology, 2022) and the NCCN “Diagnosis and Treatment Guidelines for Primary Biliary Tract Cancer (Version 2023.2)” (National Comprehensive Cancer Network, 2023) in both China and the US. The specified treatments include mFOLFOX (oxaliplatin, folinic acid and fluorouracil) for chemotherapy, pembrolizumab for immunotherapy, the anti-angiogenic inhibitor regorafenib, and a combination of irinotecan with capecitabine for other treatment options.

Drug costs were computed based on the dosing regimens in the KEYNOTE-966 trial. Drug prices for China and the US were sourced from Yaozh.com (https://www.yaozh.com/) and the Centers for Medicare & Medicaid Services (CMS) (https://www.cms.gov/), respectively. The Yaozh.com data used the average of the winning bid prices in the 6 months closest to August 2023, while CMS data utilized the average drug prices for the first half of 2023. Body surface area for Chinese and American patients was calculated based on the average height and weight reported in the “2020 China Residents’ Nutrition and Chronic Disease Status Report” and the Centers for Disease Control and Prevention (CDC) in the US Considering the prolonged course of BTC and the likelihood of weight loss in late-stage patients, the study assumed a weight reduction of 4 kg for patients. This resulted in a calculated body surface area of 1.65 m^2^ for Chinese patients and 1.93 m^2^ for American patients (National Health and Family Planning Commission, 2020; Centers for Disease Control and Prevention, 2021; Ye et al., 2023). Adverse reaction costs were referenced from literature reporting similar adverse reaction treatment costs. The cost of non-progressive CT was based on the CT price at a hospital in China (Table 1).

**TABLE 1:** Key model inputs.

Parameters	Base-case values	Ranges	Distribution	References
Cost in China ($/per cycle)				
Pembrolizumab (100 mg)	5,324.82	3,993.62–6,656.03	gamma	yaozh
Gemcitabine (1 g)	92.96	72.11–106.85	gamma	yaozh
Cisplatin (6 mL:30 mg)	9.59	7.78–11.37	gamma	yaozh
Oxaliplatin (100 mg)	133.13	89.43–148.04	gamma	yaozh
Folinic acid (100 mg)	36.64	9.70–115.56	gamma	yaozh
Fluorouracil (250 mg)	39.36	28.82–366.46	gamma	yaozh
Regorafenib (40 mg)	1724.94	1,614.69–1834.56	gamma	yaozh
Irinotecan (5 mL:0.1 g)	338.62	39.78–1,173.71	gamma	yaozh
Capecitabine (150 mg)	36.81	27.86–55.72	gamma	yaozh
Expenditures on main AEs, $				
Neutropenia	354.00	265.50–442.50	gamma	Yang et al. (2021)
Anemia	213.32	159.99–266.65	gamma	Xu et al. (2023)
Thrombocytopenia	1,054.22	790.67–1,317.78	gamma	Wen et al. (2021)
Leukopenia	495.74	371.81–619.68	gamma	Xu et al. (2023)
Cost in the US($/per cycle)				
Pembrolizumab (1 mg)	10,933.10	10,782.80–11083.40	gamma	CMS
Gemcitabine (200 mg)	69.61	67.59–71.62	gamma	CMS
Cisplatin (10 mg)	23.45	16.28–30.61	gamma	CMS
Oxaliplatin (0.5 mg)	35.69	33.47–37.90	gamma	CMS
Folinic acid (50 mg)	82.11	69.02–95.20	gamma	CMS
Fluorouracil (500 mg)	35.23	29.67–40.79	gamma	CMS
Regorafenib (40 mg)	2018.52	1,569.33–2467.71	gamma	CMS
Irinotecan (20 mg)	67.26	56.28–78.24	gamma	CMS
Capecitabine (150 mg)	226.12	144.82–307.41	gamma	CMS
Expenditures on main AEs, $				
Neutropenia	17,017	12,762.75–21271.25	gamma	Yang et al. (2021)
Anemia	81,991	61,493.25–102488.75	gamma	Xu et al. (2023)
Thrombocytopenia	9,191	6,893.25–11488.75	gamma	Wen et al. (2021)
Leukopenia	11,648	8,736–14560	gamma	Xu et al. (2023)
Image costs($/per cycle)				
No progression	45.27	33.95–56.59	gamma	local charge
Utility values				
PFS	0.79	0.60–0.80	beta	Thompson-Coon et al. (2010)
PD	0.69	0.45–0.72	beta	Thompson-Coon et al. (2010)
Disutility values				
Neutropenia	−0.2	−0.25–0.15	beta	Nafees et al. (2017)
Anemia	−0.09	−0.11–0.07	beta	Beusterien et al. (2010)
Thrombocytopenia	−0.108	−0.13–0.08	beta	Tolley et al. (2013)
Leukopenia	−0.2	−0.25–0.15	beta	Nafees et al. (2017)
Probability of AEs in pembrolizumab group				
Neutropenia	0.47	0.35–0.59	beta	Kelley et al. (2023)
Anemia	0.24	0.18–0.30	beta	Kelley et al. (2023)
Thrombocytopenia	0.16	0.12–0.20	beta	Kelley et al. (2023)
Leukopenia	0.12	0.09–0.15	beta	Kelley et al. (2023)
Probability of AEs in the chemotherapy group				
Neutropenia	0.46	0.35–0.58	beta	Kelley et al. (2023)
Anemia	0.25	0.19–0.31	beta	Kelley et al. (2023)
Thrombocytopenia	0.18	0.14–0.23	beta	Kelley et al. (2023)
Leukopenia	0.09	0.07–0.11	beta	Kelley et al. (2023)
Probability of follow-up treatment in the pembrolizumab group				
Chemotherapy	0.75	0.56–0.94	beta	Kelley et al. (2023)
Immunotherapy	0.09	0.07–0.11	beta	Kelley et al. (2023)
Targeted Therapy	0.02	0.02–0.03	beta	Kelley et al. (2023)
Other	0.14	0.11–0.18	beta	Kelley et al. (2023)
Probability of follow-up treatment in the chemotherapy group				
Chemotherapy	0.69	0.52–0.86	beta	Kelley et al. (2023)
Immunotherapy	0.11	0.08–0.14	beta	Kelley et al. (2023)
Targeted Therapy	0.05	0.04–0.06	beta	Kelley et al. (2023)
Other	0.15	0.11–0.19	beta	Kelley et al. (2023)

AEs, adverse events; US, United States; PFS, progression-free survival; PD, progressed disease.

### Utility

The KEYNOTE-966 trial did not disclose the health utility values of patients, and currently available data also lack health utility values for BTC, therefore, reference must be made to the health utility values of other cancer types. This study adopts the health utility values for renal cell carcinoma (PFS: 0.79, PD: 0.69) (Thompson-Coon et al., 2010), and negative utility values for AEs are referenced from the same adverse reaction values in advanced chronic lymphocytic leukemia, assuming that leukopenia has the same utility value as neutropenia (Beusterien et al., 2010; Tolley et al., 2013; Nafees et al., 2017).

### Sensitivity analysis

The robustness of the model depends on the ICER values, and parameter changes cause changes in the ICER values. This study conducted sensitivity analyses on the parameters used to calculate the ICER, including one-way sensitivity analysis and probabilistic sensitivity analysis. One-way sensitivity analysis adjusts the value of a parameter variable. If there is no definite value range, the data within the range of ±25% are used in the analysis to obtain the maximum and minimum ICER values, which are then compared with the WTP. If the conclusion of the one-way sensitivity analysis is consistent with that of the basic analysis, it means that the change in this variable will not cause fundamental changes to the fundamental analysis and has good stability. Probabilistic sensitivity analysis can simultaneously consider the impact of changes in multiple uncertainties on the outcome. A total of 1,000 simulations were performed using the second-order Monte Carlo simulation, with the gamma distribution used for the cost parameters and the beta distribution used for the health utility values and incidence (Briggs et al., 2006). Incremental cost-effectiveness scatter plots and cost-effectiveness acceptability curves were generated from simulation results to assess the economic probability of the two interventions at different WTP thresholds.

## Results

### Basic analysis

**FIGURE 2:**
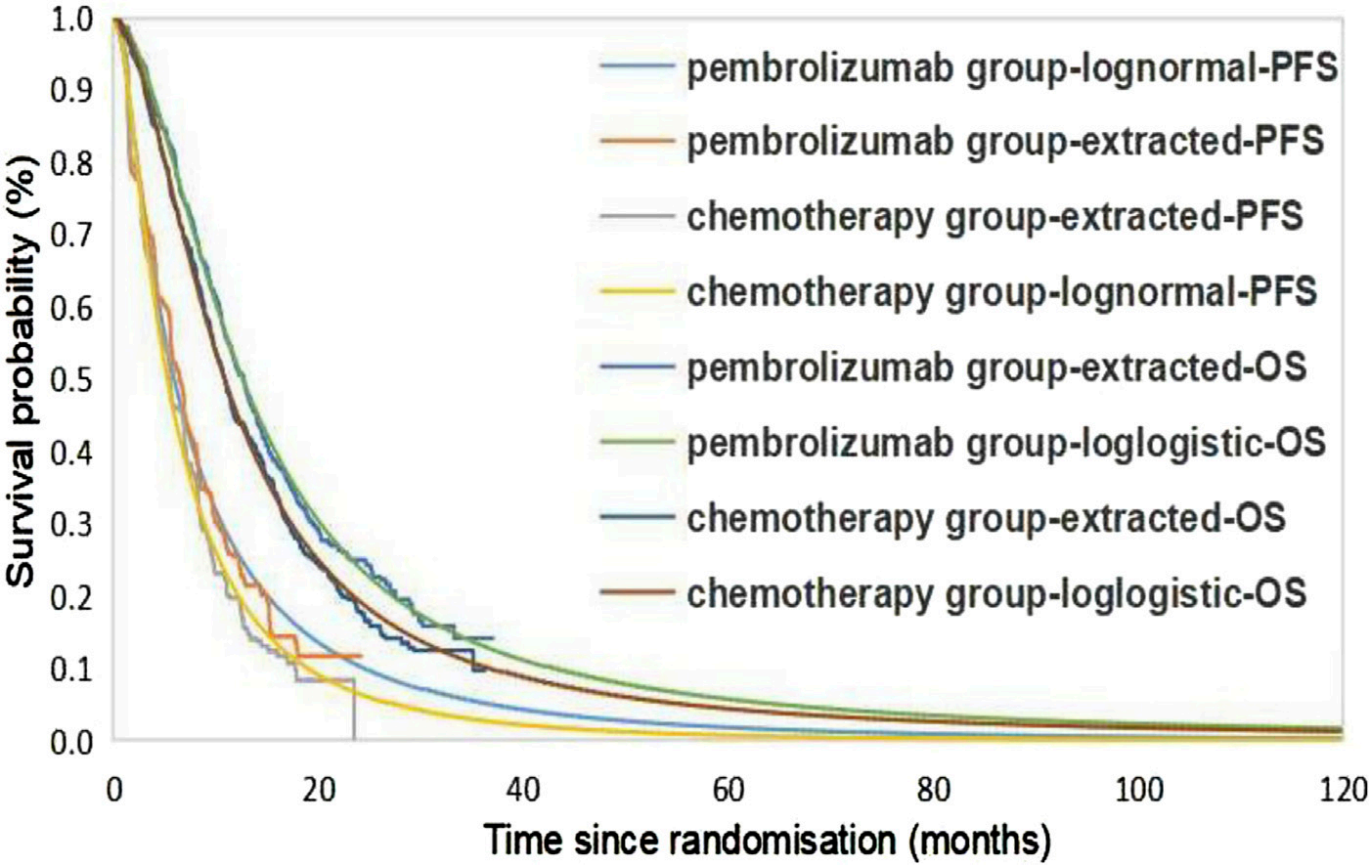
Kaplan-Meier Cure in the pembrolizumab and chemotherapy group using standard parametric models fitting and extrapolation. PFS, progression-free survival; OS, overall survival.

The extrapolation curves for PFS in both the pembrolizumab group and the placebo group best fit a lognormal distribution, while the extrapolation curves for OS in both groups best fit a log-logistic distribution (see Table 2 for curve-fitting parameters; Figure 2 for fitted curves). From the perspective of the Chinese healthcare system, the pembrolizumab group yields 1.55 LYs and 1.15 QALYs at a total cost of $88,744.90; the placebo group yields 1.37 LYs, 1.01 QALYs, with a corresponding total cost of $11,629.96. The additional cost of pembrolizumab compared to placebo is $77,114.94 and the additional QALYs was 0.14, resulting in an ICER of $556,689.47/QALY, significantly exceeding the current WTP threshold in China ($38,201.19), indicating that the use of pembrolizumab in combination with chemotherapy for first-line treatment of BTC is not economically viable at present prices. From the perspective of the US healthcare system, the additional cost of the pembrolizumab group compared to the chemotherapy group is $160,425.24, and the additional QALYs was 0.14, with an ICER of $1,109,462.92/QALY, also significantly higher than the US WTP (see Table 3).

**TABLE 2:** Clinical Inputs: Kaplan-Meier survival curves Fitting Parameters.

	Best fitting	Parameters	AIC	BIC
Pembrolizumab PFS	Lognormal	mean = 1.7765	2300.656	2309.213
		sd = 1.1090		
Pembrolizumab OS	Loglogistic	shape = 1.7984	3,155.376	3,163.933
		scale = 12.6047		
Chemotherapy PFS	Lognormal	mean = 1.6558	2335.670	2344.238
		sd = 1.0038		
Chemotherapy OS	Loglogistic	shape = 1.8019	3,252.419	3,260.988
		scale = 10.8447		

PFS, progression-free survival; PD, progressed disease; sd, standard deviation; AIC, Akaike information criterion; BIC, Bayesian information criterion.

**TABLE 3:** Results.

Strategies	Cost	Incr cost	LYs	Incr LYs	ICER/LYs	QALYs	Incr QALYs	ICER/QALYs
Chinese health system perspective								
Pem	88,744.90	77,114.94	1.55	0.18	432,152.91	1.15	0.14	556,689.47
Chem	11,629.96		1.37			1.01		
US health system perspective								
Pem	210,344.33	160,425.24	1.59	0.19	861,070.81	1.18	0.14	1,109,462.92
Chem	49,919.09		1.41			1.03		

Incr, incremental; Lys, life years; ICER, incremental cost-effectiveness ratio; QALYs, quality-adjusted life years; Pem, pembrolizumab; Chem, chemotherapy.

### One-way sensitivity analysis

**FIGURE 3:**
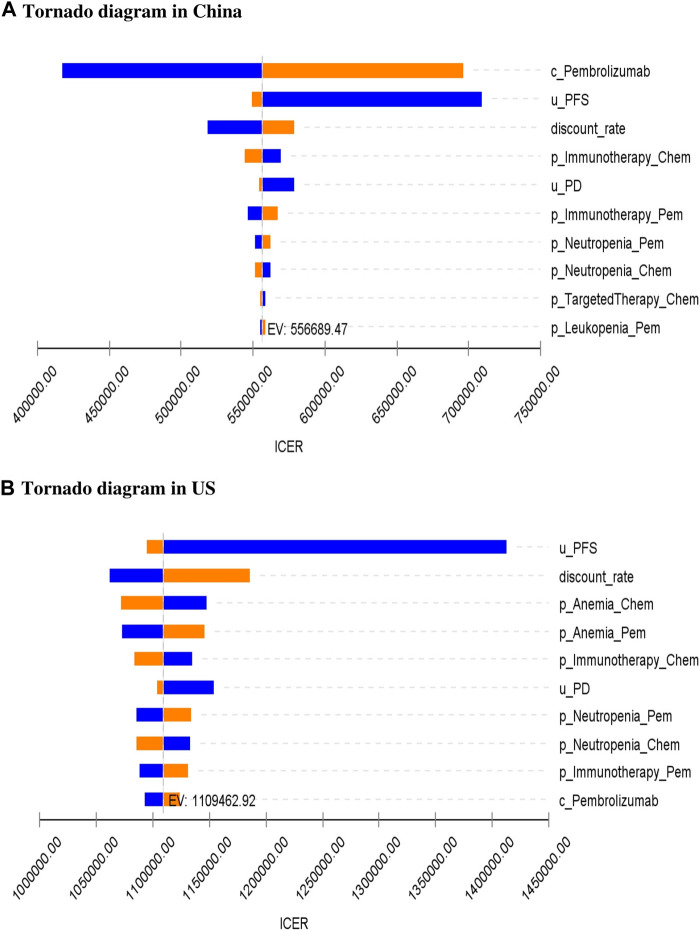
One-way sensitivity analysis for pembrolizumab and chemotherapy group. c, cost; u, utility; p, probability; PFS, progression-free survival; PD, progressed disease; OS, overall survival; Chem, chemotherapy; Pem, pembrolizumab; ICER, incremental cost-effectiveness ratio; EV, expected value; US, United States.

As can be seen from the tornado diagram from the perspective of the Chinese health system (Figure 3A), the parameters that had the greatest impact on the results of the base analysis are the price of pembrolizumab, the utility value for PFS, and the discount rate. A tornado diagram from the perspective of the US health system (Figure 3B) reveals that the utility value for PFS, discount rate, and probability of occurring anemia in both experimental groups have the greatest impact on ICER outcomes. However, whether using the maximum or minimum values for these variables, the sensitivity analysis results remain consistent with the base-case analysis, consistently showing an ICER higher than the WTP threshold.

### Probabilistic sensitivity analysis

**FIGURE 4:**
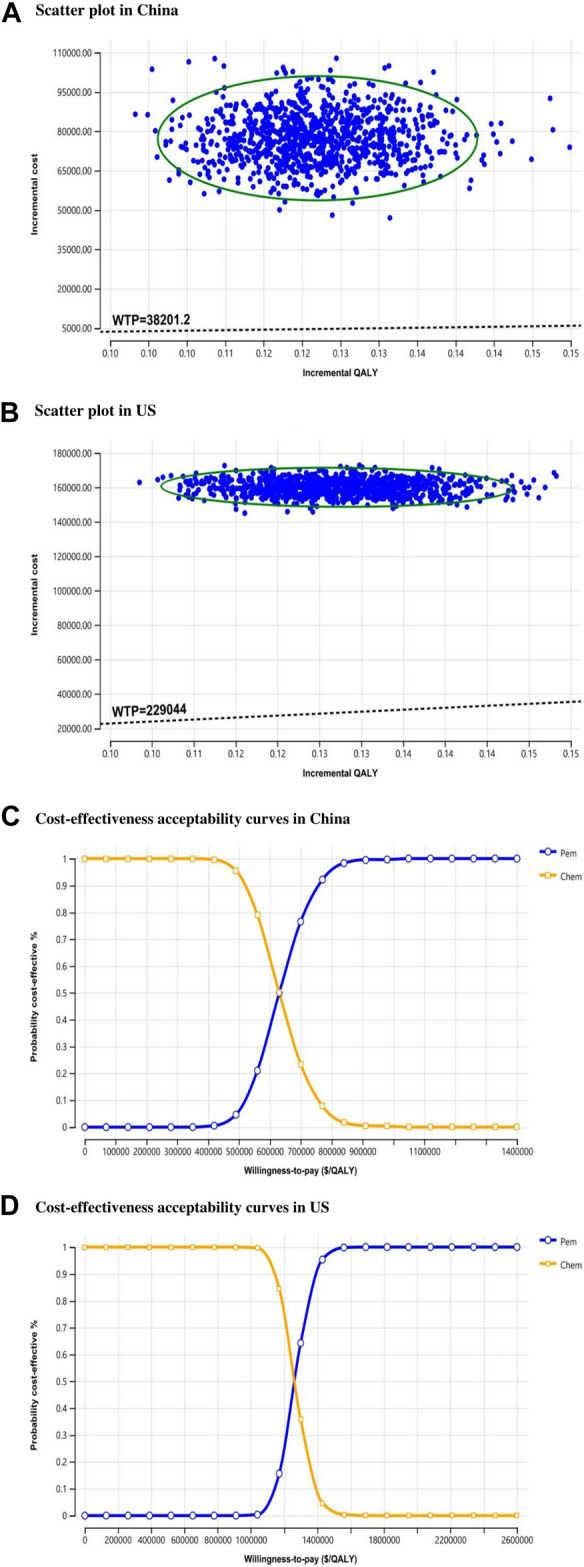
Probability sensitivity analysis. Scatter plot comparing pembrolizumab and chemotherapy group. WTP, willing-to-pay; QALY, quality-adjusted life year; US, United States; Chem, chemotherapy; Pem, pembrolizumab.

The probabilistic sensitivity analysis assesses the overall impact on results when each parameter is assigned a specific value. The ICER scatter plots from the perspective of the Chinese healthcare system (Figure 4A) reveal that all points are distributed above the WTP threshold. This pattern is also observed in the ICER scatter plots from the US healthcare system perspective (Figure 4B). Cost-effectiveness acceptability curves (Figure 4C) indicate that at China’s current WTP, the probability of pembrolizumab plus chemotherapy being cost-effective is 0%. To achieve a 100% probability of economic benefit, the WTP would need to approach nearly $900,000. Figure 4D shows that for pembrolizumab in combination with chemotherapy to have a 100% probability of economic benefit in the US, the WTP would need to be close to $1.6 million.

## Discussion

Due to the differences in national conditions and healthcare environments, we conducted an economic evaluation from the perspectives of both China and the US. Our study results can provide valuable economic information for the use of pembrolizumab in treating BTC. Based on the base-case estimates of pembrolizumab group versus the chemotherapy group, the ICER in China is $556,689.47/QALY and in the US is $1,109,462.92/QALY, exceeding the WTP thresholds of $38,201.19 and $229,044, respectively. Sensitivity analyses consistently demonstrate the robustness of the model. These results indicate that whether in China or the US, pembrolizumab is unlikely to be a cost-effective method for treating advanced BTC. One-way sensitivity analysis in China reveals that the price of pembrolizumab is the most influential factor in the study. The analysis shows that from the perspective of the Chinese healthcare system, the ICER consistently exceeds the WTP threshold, with pembrolizumab becoming cost-effective only when its price is reduced by 91%, for example, to 9% ($207.67/100_mg) of the original price, resulting in an ICER of $37,990.34/QALY. In the US, one-way sensitivity analysis indicates that drug price is not a significant factor affecting ICER results, but when pembrolizumab is discounted by 79% ($12.58/mg), the ICER is reduced to $228,227.83/QALY, below the US WTP ($229,044), making this treatment approach cost-effective. Considering the usual reimbursement ratio of 80/20 for drug costs between insurance companies and individuals in the US, since the US Medicare reimbursement ratio is usually 80/20, the insurance company pays 80% of the cost, and the individual pays 20%, reducing the ICER in the US by 80% to $221,892.59/QALY, which is lower than the WTP in the US, offering more hope to patients. Although pembrolizumab has not yet entered the medical insurance directory in China, it is fortunate that the donation project “Key to Life” of pembrolizumab officially accepts applications from first-line treatment indications of pembrolizumab combined with gemcitabine and cisplatin for locally advanced or metastatic BTC patients who meet the project conditions starting from 20 February 2024. According to the plan provided by the assistance project, the cost of pembrolizumab group after receiving assistance was calculated, and the results showed a cost reduction of $48,868.83. The ICER result was $203,907.50/QALY, which is higher than the WTP in China, but compared to the ICER ($556,689.47/QALY) when no assistance was received, it has already saved half of the cost.

The above results indicate that reducing the price of pembrolizumab is crucial to enhance its feasibility as a preferred treatment option. Pembrolizumab has obtained approval for 10 indications in China and 32 indications in the US, with a large patient population and a vast market. Appropriate price reduction can increase sales while saving more lives. Currently, both the Chinese and US governments have taken actions to lower the prices of anticancer drugs. For instance, as part of healthcare reform, the Chinese government initiated a centralized drug procurement plan, leading to a significant decrease in prices for many drugs on the procurement list. In the US, Medicare engages in direct negotiations with pharmaceutical companies under the Inflation Reduction Act (IRA). These government measures aim to reduce drug prices, alleviate financial pressure on government healthcare, enable more patients to access innovative treatments, and improve survival rates. Therefore, substantial price reductions or financial support are crucial for patients to access innovative treatments.

In the systemic treatment regimen for BTC, although the safety of ICIs monotherapy and immune-based combination therapy is acceptable, ICIs have a specific set of treatment-related adverse events that may affect multiple organ systems, including the liver, thyroid, lung, pancreas, and skin (Marrone et al., 2016; Rizzo et al., 2021). Hepatotoxicity is often underestimated due to unclear clinical presentation and low incidence compared with other common adverse events. Given the increased incidence of these toxicities, monitoring of liver function should be recommended for cancer patients receiving ICIs monotherapy or immune-based combination therapy (Rini et al., 2022; Rizzo et al., 2023).

The potential value of the results of this study lies in their use as a reference for future medical decisions such as the drug price adjustment of pembrolizumab, the reimbursement of medical insurance in the United States, and the inclusion of pembrolizumab in the Chinese medical insurance list. The pharmacoeconomic evaluation on which this study is based is more likely to favor the decision-making analysis of the healthcare sector or different stakeholder groups in the future, as well as real-world data, precision medicine and digital health based on it.

The limitations of this article include, firstly, the unavailability of follow-up imaging prices in the United States, necessitating the use of prices in China for calculation. It is hoped that future availability of US imaging price data will supplement and improve this study. Secondly, due to the absence of reported quality of life or utility data in the KEYNOTE-966 trial, the utility data of other tumors are temporarily referenced in view of other pharmacoeconomic evaluation methods for oncology, and the utility values in China are assumed to be the same as those in the US. However, a sensitivity analysis was conducted within the range of variation in utility values to explore the variations of results. The results showed that a change in utility values within this range had no effect on the conclusions. In addition, the KEYNOTE-966 trial reports and annexes on subsequent follow-up for BTC progression were only available for the proportion of different treatment categories, with no specific treatment options for those categories available, the protocol for follow-up in this study was derived from consulting specialists and reviewing pharmacoeconomic literature on other BTC. Lastly, the inability to access individual patient data and the calculation of transition probabilities between different states based on fitting reported Kaplan-Meier PFS and OS curve data may introduce uncertainty into the model outputs.

## Conclusion

This study, employing a Markov model approach, investigates the cost-effectiveness of pembrolizumab in combination with chemotherapy for the treatment of advanced BTC from the perspectives of the healthcare systems in China and the US. The results indicate that the pembrolizumab plus chemotherapy regimen offers higher survival benefits compared to standard chemotherapy. However, both in China and the US, the combination therapy with pembrolizumab is not cost-effective as a first-line treatment for advanced BTC. Appropriate price reduction of pembrolizumab would enhance its economic feasibility.

## Data Availability

The original contributions presented in the study are included in the article/Supplementary Material, further inquiries can be directed to the corresponding authors.
