# Supplementary material for: Cost-effectiveness of pembrolizumab plus chemotherapy vs. chemotherapy as first-line treatment for advanced biliary tract cancer in China and the US
Source: Front Pharmacol. 2024 Aug 14;15:1393559. doi: 10.3389/fphar.2024.1393559 (PMC11349542; doi:10.3389/fphar.2024.1393559)
Supplement: Supplementary file 1 [file DataSheet1.PDF]

Supplementary Material

Cost-effectiveness of pembrolizumab plus chemotherapy as first-line treatment for advanced biliary tract cancer in China and the US

Xianmei Luo[1,2✦], Tingting Cai[1,2✦], Jinyan Wu[3], Xingyu Li[1,2], Xiaofan Wang[1,2*], Haiying Ma[1,2*]

1 Department of Pharmacy, The Fourth Affiliated Hospital of China Medical University, Shenyang, China
2 School of Pharmacy, China Medical University, Shenyang, China
3 Key Laboratory of Birth Defects and Related Diseases of Women and Children of MOE, State Key Laboratory of Biotherapy, West China Second University Hospital, Sichuan University, Chengdu, China

[✦]These authors contributed equally to this work.

[*] Correspondence:
Xiaofan Wang, Haiying Ma
E-mail: xfwang@cmu.edu.cn, cmu4h-mhy@126.com

Content

Table S1 Baseline characteristics of patients in KEYNOTE-966 3
Table S2 Adverse reactions with a grade ≥ 3 and an incidence of ≥ 9% 4
Table S3 Comparison of fitting results 5
Table S4 Standard parametric model 6
Figure S1 Contrast between original curve and extracted curve 7
Figure S2 AIC and BIC value visualization 9
Figure S3 Survival plots showing the goodness-of-fit 13
Figure S4 Pembrolizumab price simulation 17
Method 18
1. Treatment options 18
2. Supplementary information of key exclusion criteria from KEYNOTE-966 19
3. Half- Cycle Correction in TreeAge Pro 20
4. The method of calculating the transfer probability of PD status 21
Reference 22

Table S1 Baseline characteristics of patients in KEYNOTE-966

	Pembrolizumab(n=533)	Placebo(n = 536)
Age, years	64.0 (57.0–71.0)	63.0 (55.0–70.0)
<65	269 (50%)	298 (56%)
≥65	264 (50%)	238 (44%)
Sex		
Female	253 (47%)	264 (49%)
Male	280 (53%)	272 (51%)
Geographical region		
Asian	242 (45%)	244 (46%)
Non-Asian	291 (55%)	292 (54%)
ECOG performance status		
0	258 (48%)	228 (43%)
1	274 (51%)	308 (57%)
≥2	1 (<1%)	0
Site of origin		
Extrahepatic	98 (18%)	105 (20%)
Gallbladder	115 (22%)	118 (22%)
Intrahepatic	320 (60%)	313 (58%)
Hepatitis B status		
Any viral hepatitis B*	164 (31%)	165 (31%)
Chronic infection	14 (3%)	16 (3%)
Clinically resolved infection	150 (28%)	149 (28%)
No viral hepatitis B	366 (69%)	366 (68%)
Missing	3 (1%)	5 (1%)
Hepatitis C status		
Any viral hepatitis C†	19 (4%)	14 (3%)
Active infection	1 (<1%)	1 (<1%)
Previous infection	18 (3%)	13 (2%)
No viral hepatitis C	514 (96%)	520 (97%)
Missing	0	2 (<1%)
Microsatellite instability status		
Microsatellite instability high	6 (1%)	4 (1%)
Microsatellite stable	433 (81%)	422 (79%)
Unknown	94 (18%)	110 (21%)
PD-L1 combined positive score		
<1	113 (21%)	110 (21%)
≥1	363 (68%)	365 (68%)
Unknown	57 (11%)	61 (11%)

Note: Content of this table reflected the baseline characteristics of patients with advanced biliary tract cancer in the KEYNOTE-966 trial. ECOG：Eastern Cooperative Oncology Group. *Chronic hepatitis B infection included participants positive for HBsAg or who had hepatitis B DNA ≥20 IU/mL. †Chronic hepatitis C infection included participants positive for hepatitis C IgG antibody and a numerical value for hepatitis C virus RNA.

Table S2 Adverse reactions with a grade ≥ 3 and an incidence of ≥ 9%

	Pembrolizumab plus gemcitabine and cisplatin group (n=529)			Placebo plus gemcitabine and cisplatin group (n=534)		
	**Grade 3**	**Grade 4**	**Grade 5**	**Grade 3**	**Grade 4**	**Grade 5**
Neutro-	158 (30%)	89	0	167 (31%)	79 (15%)	0
Anemia	122 (23%)	1 (<1%)	0	127 (24%)	4 (1%)	0
Thrombo-	55 (10%)	30 (6%)	0	66 (12%)	33 (6%)	0
Leukopenia	57 (11%)	4 (1%)	0	43 (8%)	3 (1%)	0

Table S2 Comparison of fitting results

Group	Number at risk	Median survival time(months)	95% Cl(months)
Original pembrolizumab group PFS	533	6.5	5·7–6·9
Reconstructed pembrolizumab group PFS	533	6.5	5.7-6.9
Original pembrolizumab group OS	533	12.7	11·5–13·6
Reconstructed pembrolizumab group OS	533	12.7	11.5-13.9
Original chemotherapy group PFS	536	5.6	5·1–6·6
Reconstructed chemotherapy group PFS	536	5.6	5.2-6.7
Original chemotherapy group OS	536	10.9	9·9–11·6
Reconstructed chemotherapy group OS	536	10.9	9.9-11.7

Table S3 Standard parametric model

Distribution	Shape	Survival function
Exponential	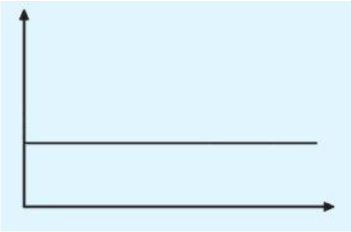	$S\ (t) = e^{-\lambda t}$
Weibull	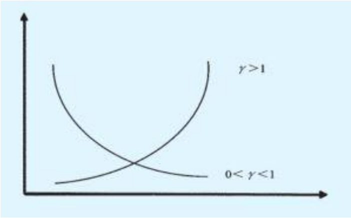	$S\ (t) = exp(-\lambda t^{\gamma})$
Gompertz	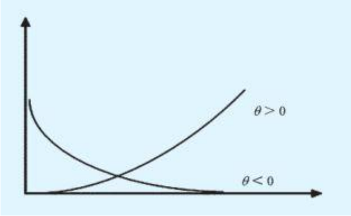	$S\ (t) = exp\left\{\frac{\lambda}{\theta}\left(1 - e^{\theta t}\right)\right\}$
Log-logistic	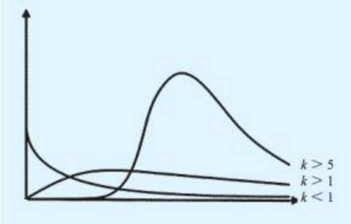	$S\ (t) = \left(1 + e^{\theta} t^{K}\right)^{-1}$
Lognormal	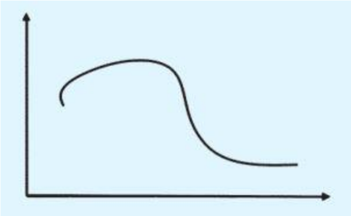	$S\ (t) = 1 - \Phi\left(\frac{\log t - \mu}{\delta}\right)$
Generalised gamma	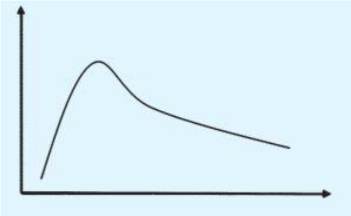	$1 - S\ (t) = 1 - \Gamma(\lambda t)\theta(\rho)$

Figure S1 Contrast between original curve and extracted curve

OS: overall survival; PFS: progression-free survival

A Original PFS curve

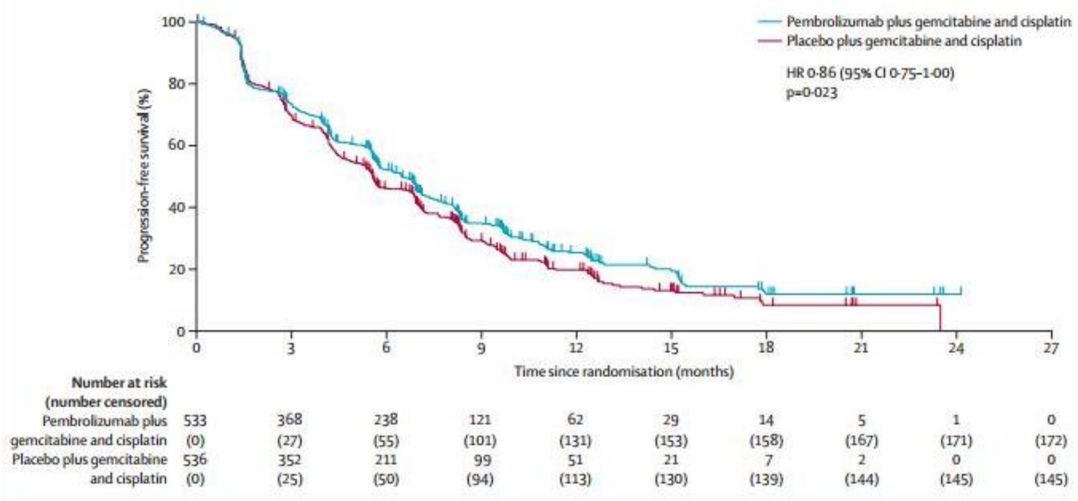

B Extracted PFS curve

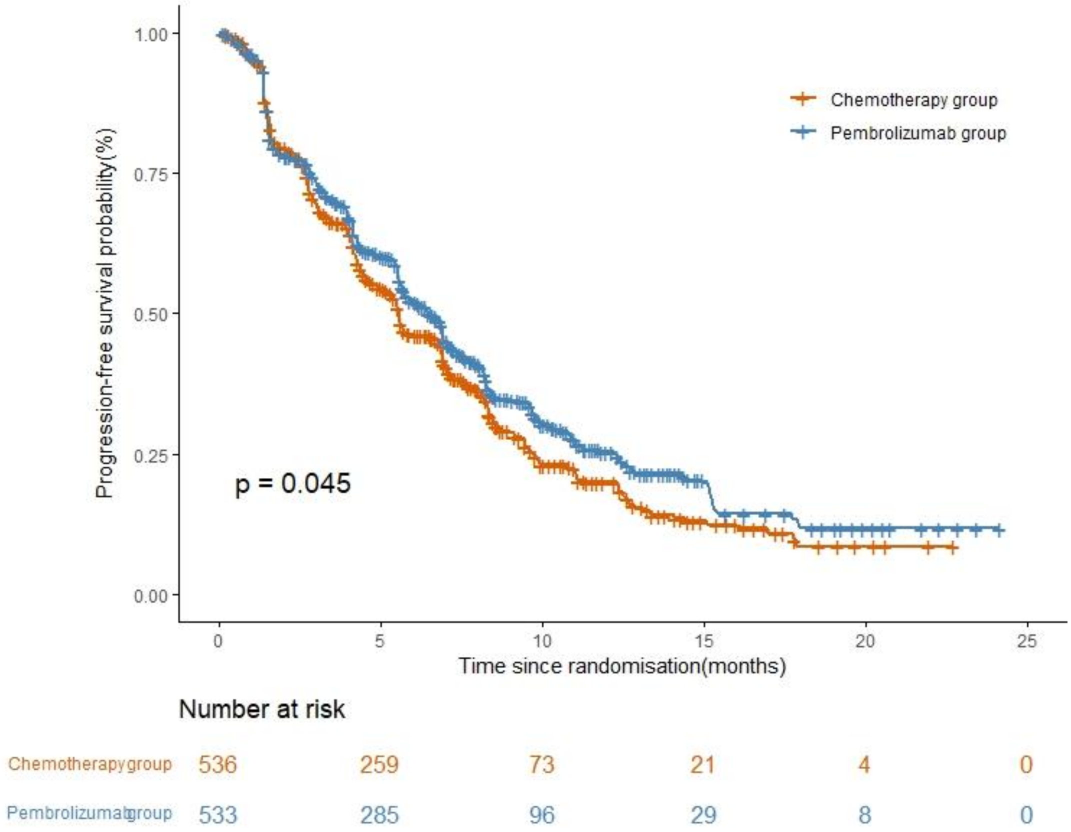

C Original OS curve

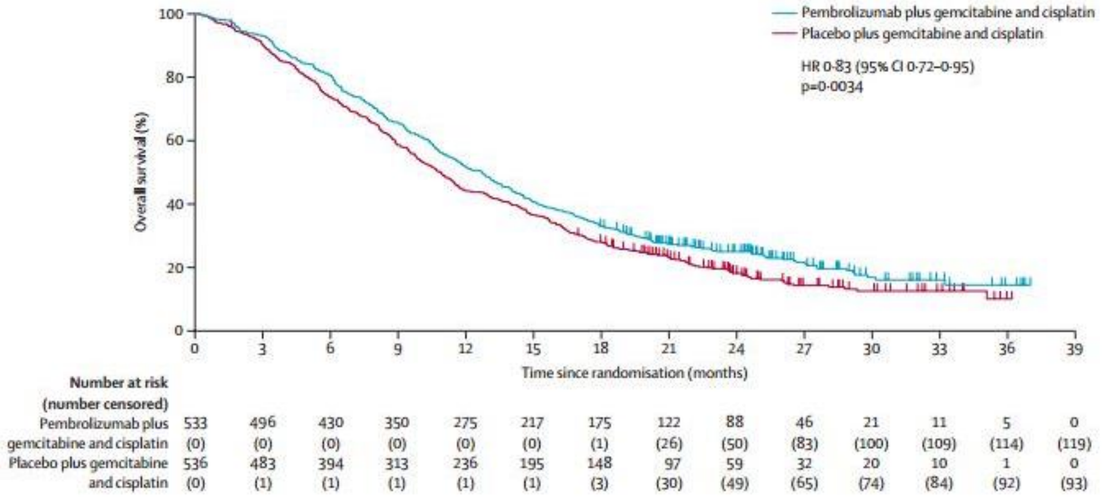

D Extracted OS curve

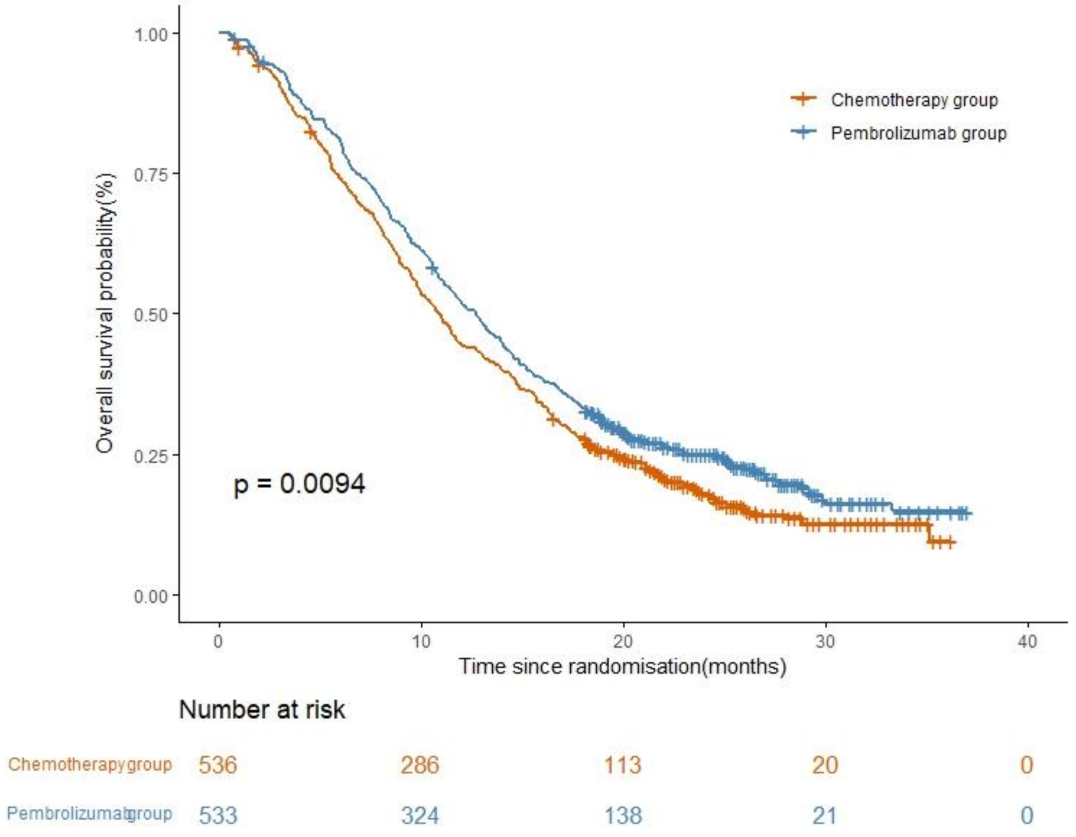

Figure S2 AIC and BIC value visualization

AIC: Akaike information criterion; BIC: Bayesian information criterion;Pem: pembrolizumab; Chem: chemotherapy; OS: overall survival; PFS: progression-free survival

A PFS-Pem-AIC

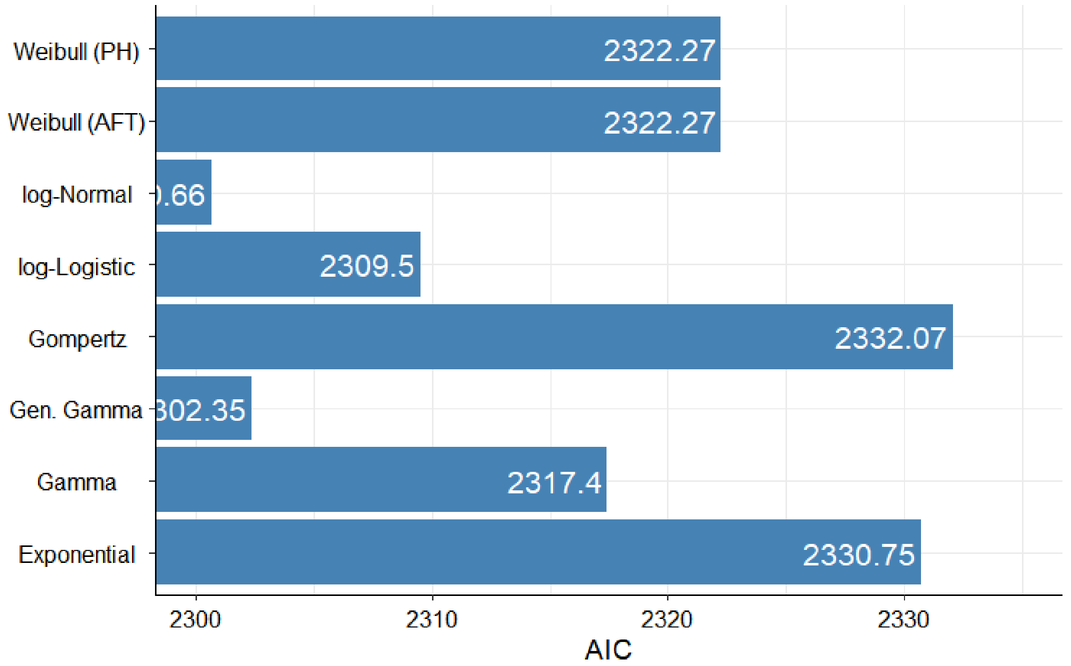

B PFS-Pem-BIC

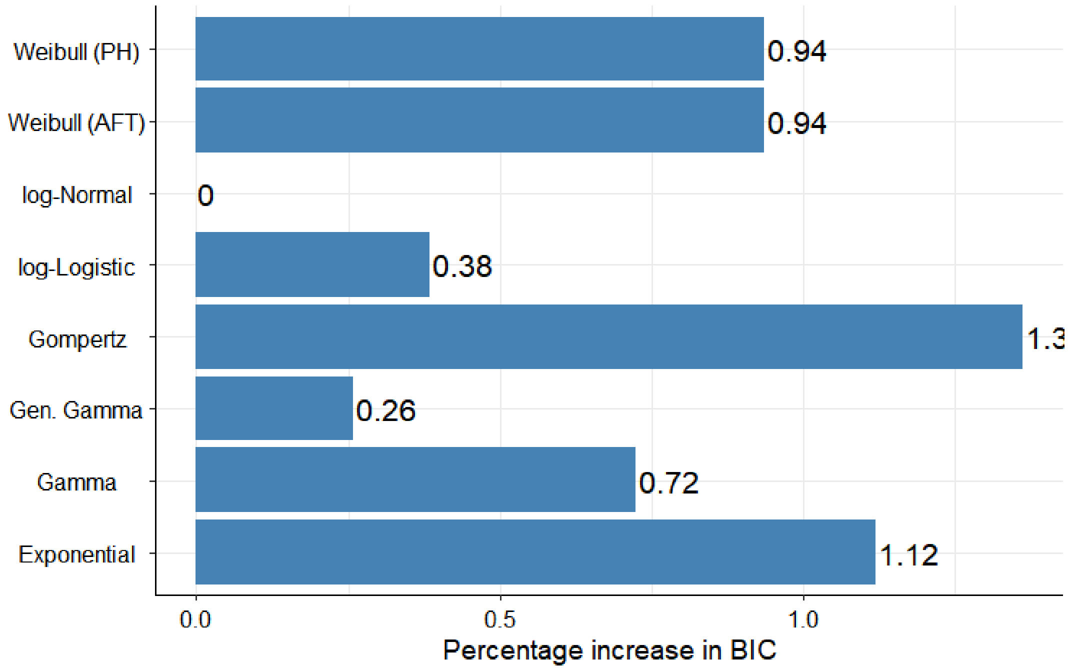

C PFS-Chem-AIC

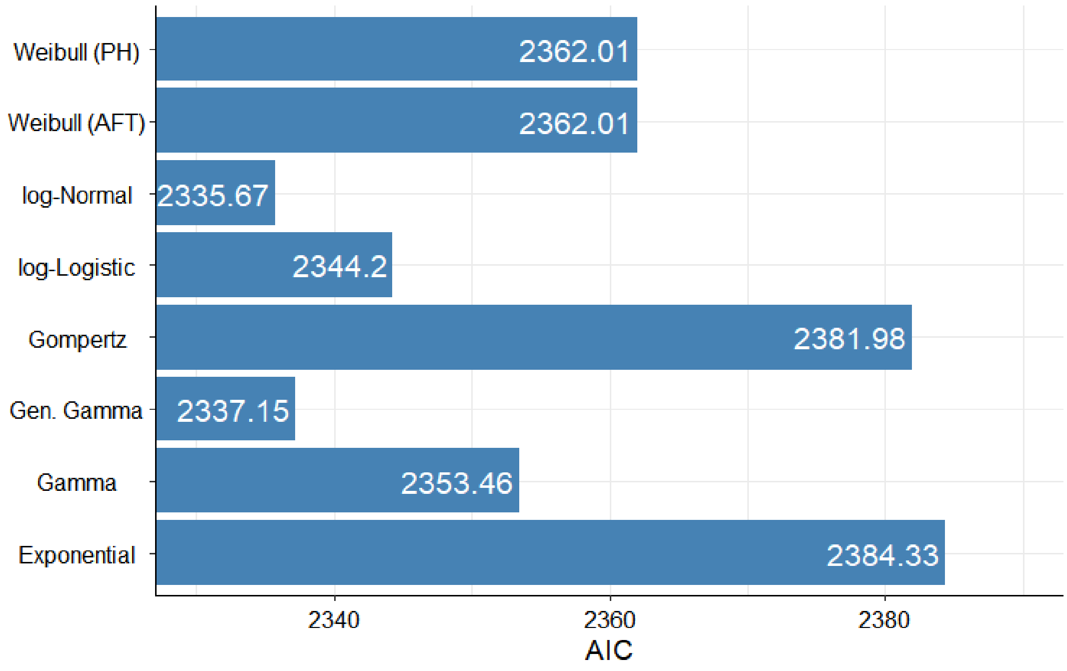

D PFS-Chem-BIC

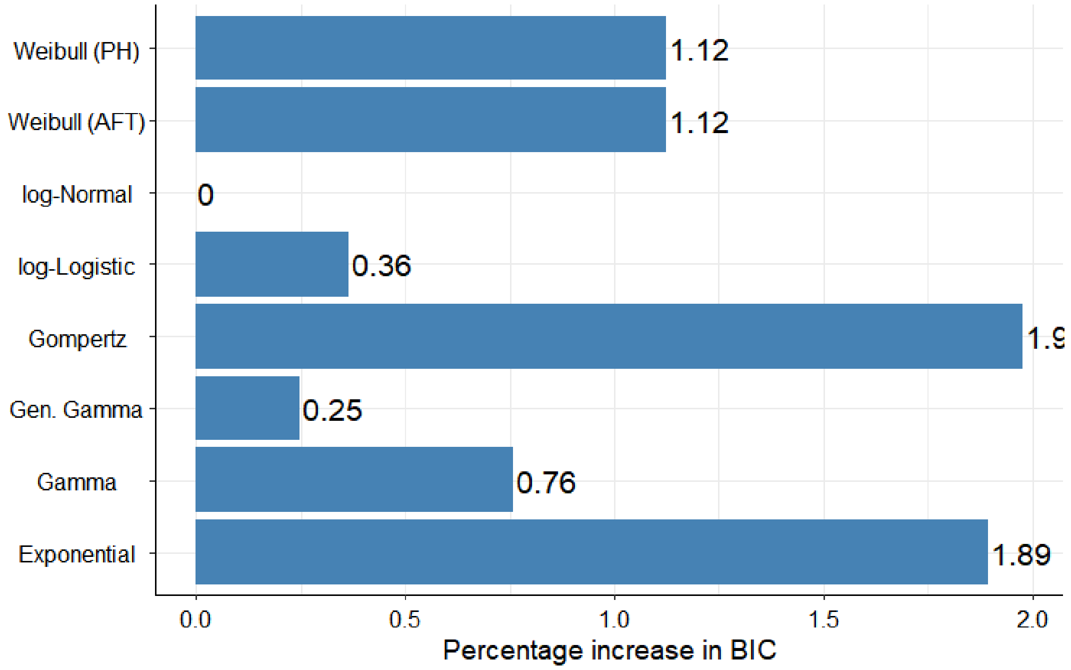

E OS-Pem-AIC

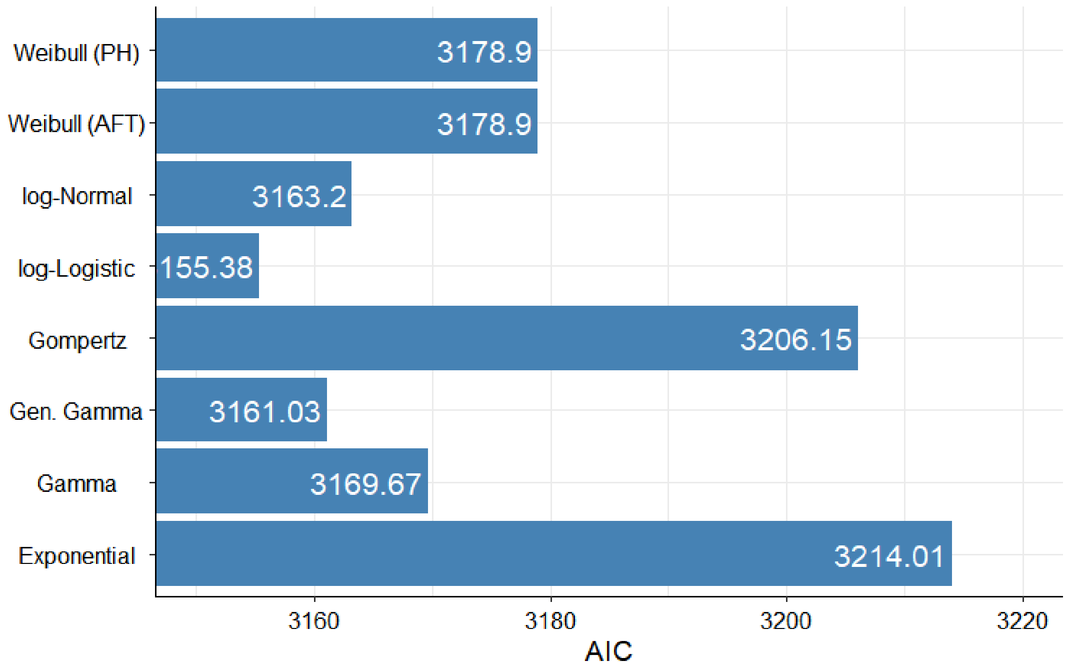

F OS-Pem-BIC

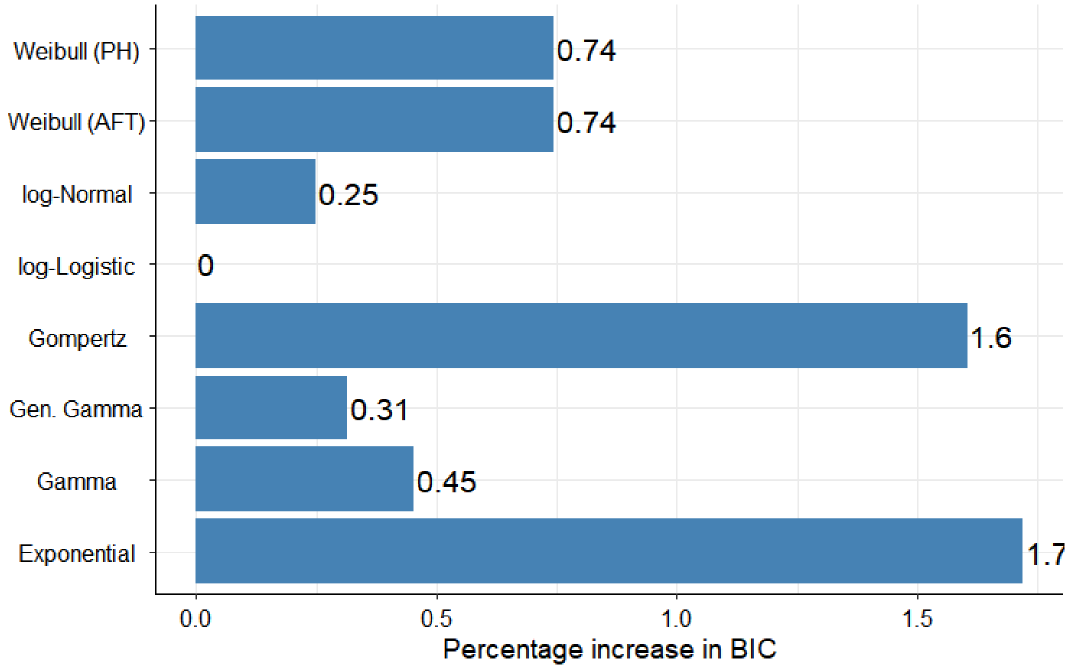

G OS-Chem-AIC

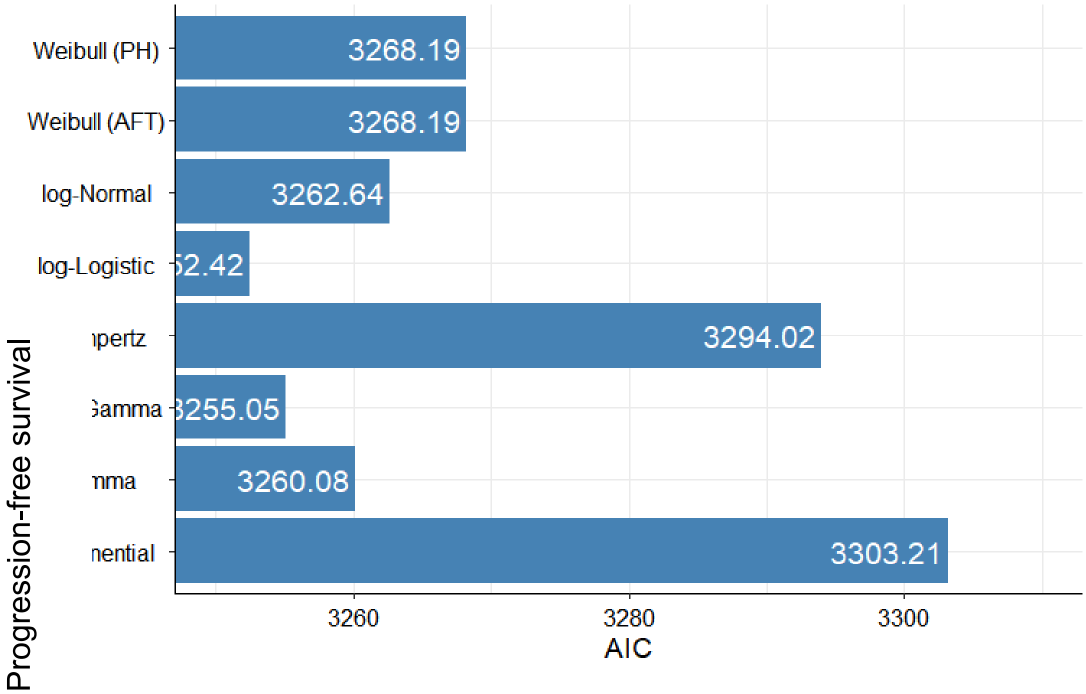

H OS-Chem-BIC

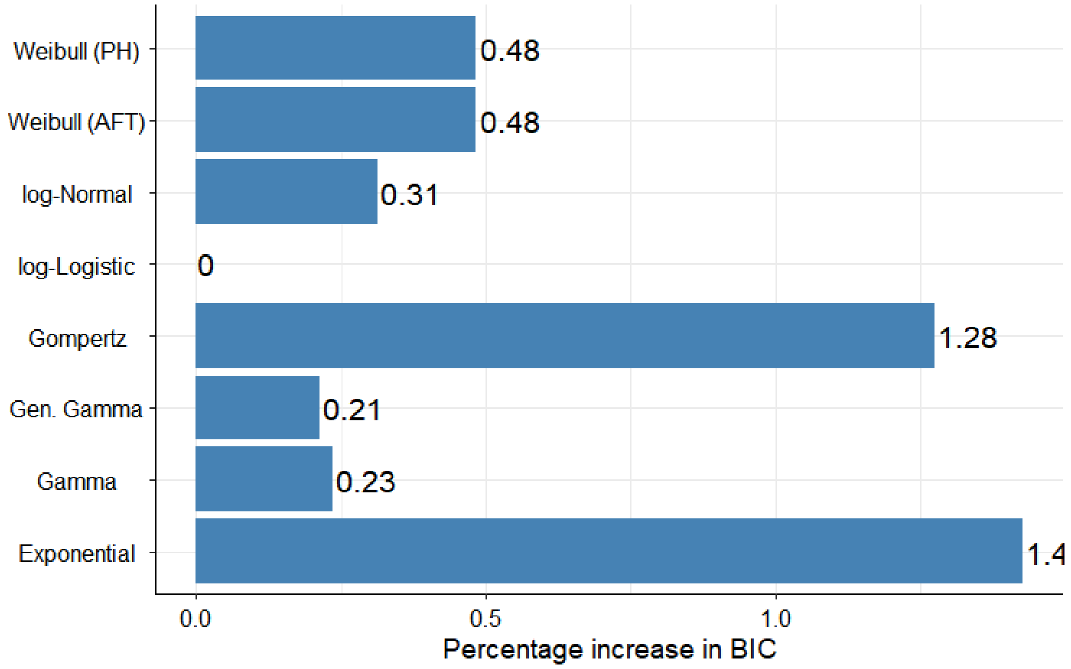

Figure S3 Survival plots showing the goodness-of-fit

Pem: pembrolizumab; Chem: chemotherapy; OS: overall survival; PFS: progression-free survival

PFS-Pem

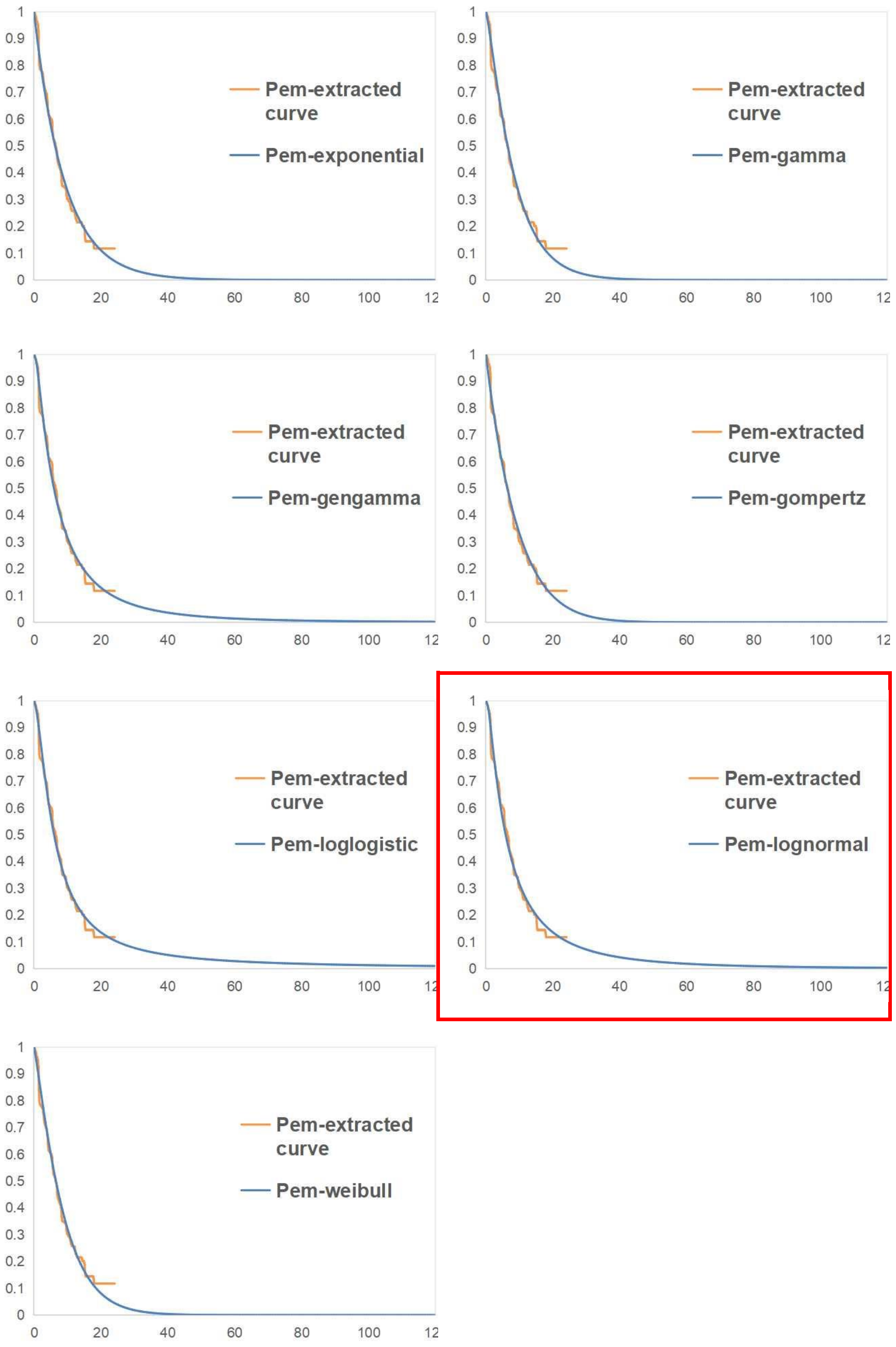

Time(years)

PFS-Chem

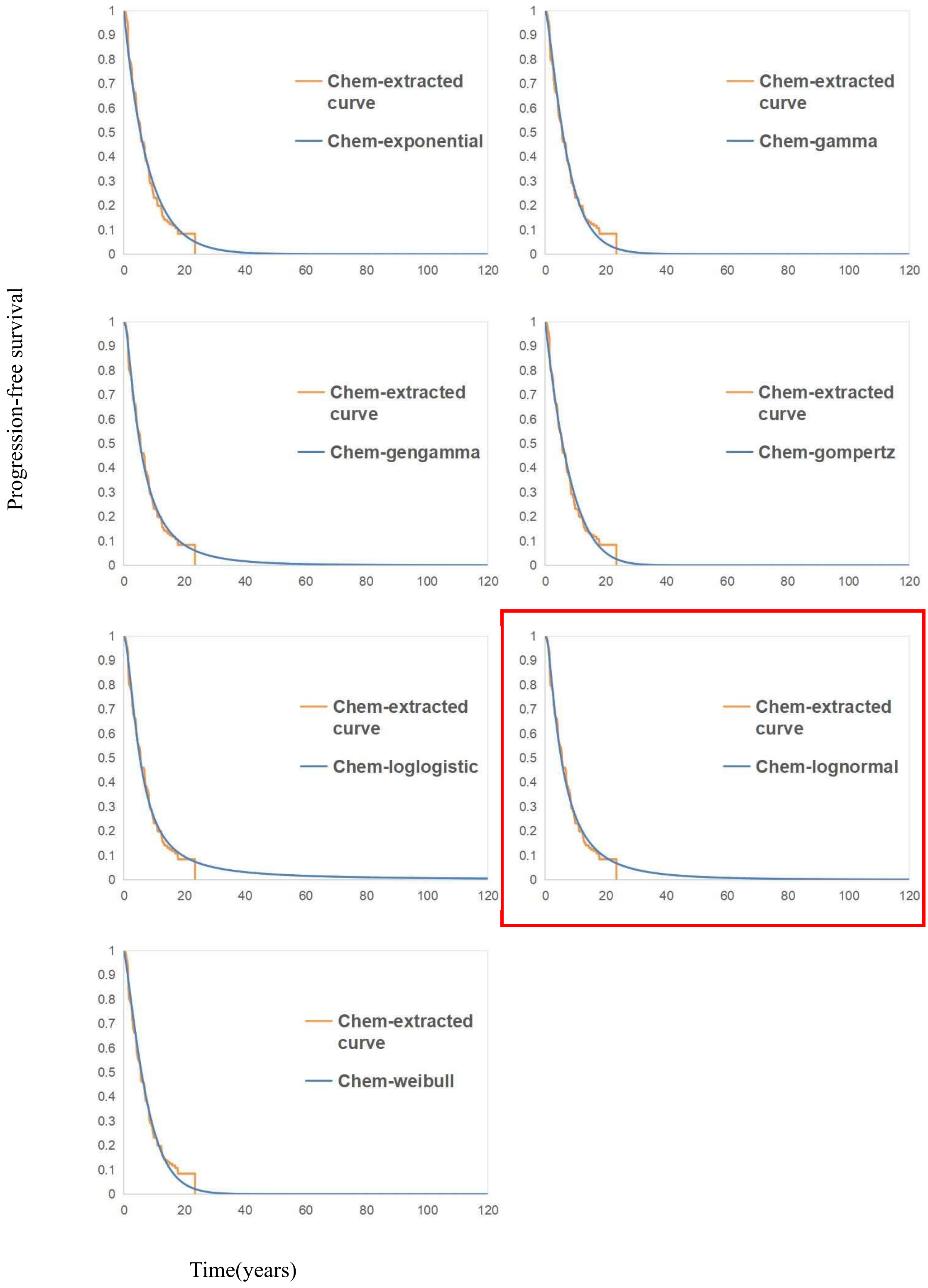
Progression-free survival
Chem-extracted curve
Chem-exponential
Chem-extracted curve
Chem-gamma
Chem-extracted curve
Chem-gengamma
Chem-extracted curve
Chem-gompertz
Chem-extracted curve
Chem-loglogistic
Chem-extracted curve
Chem-lognormal
Chem-extracted curve
Chem-weibull
Time(years)

OS-Pem

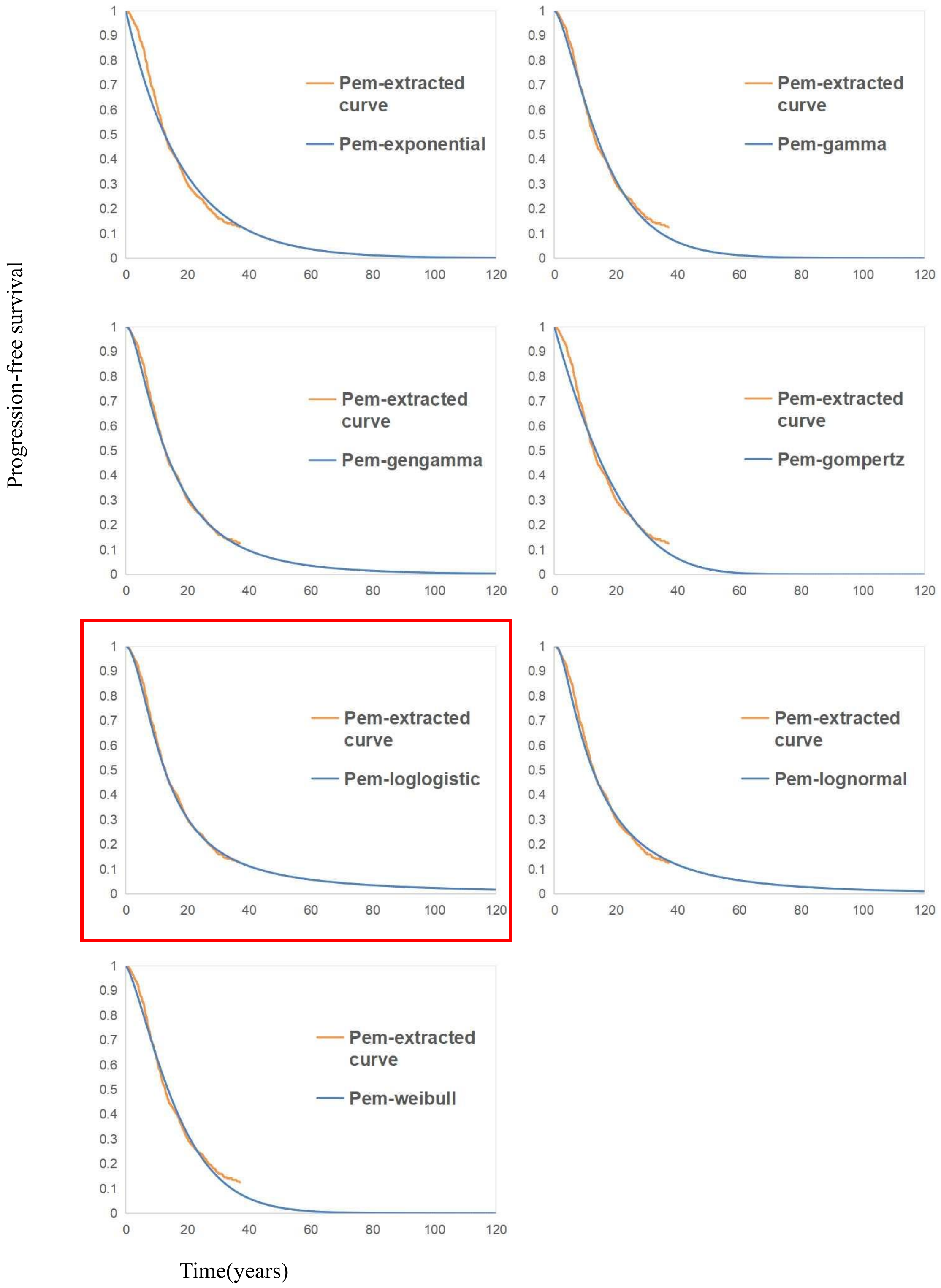

Progression-free survival
Pem-extracted curve
Pem-exponential
Pem-extracted curve
Pem-gamma
Pem-extracted curve
Pem-gengamma
Pem-extracted curve
Pem-gompertz
Pem-extracted curve
Pem-loglogistic
Pem-extracted curve
Pem-lognormal
Pem-extracted curve
Pem-weibull
Time(years)

OS-Chem

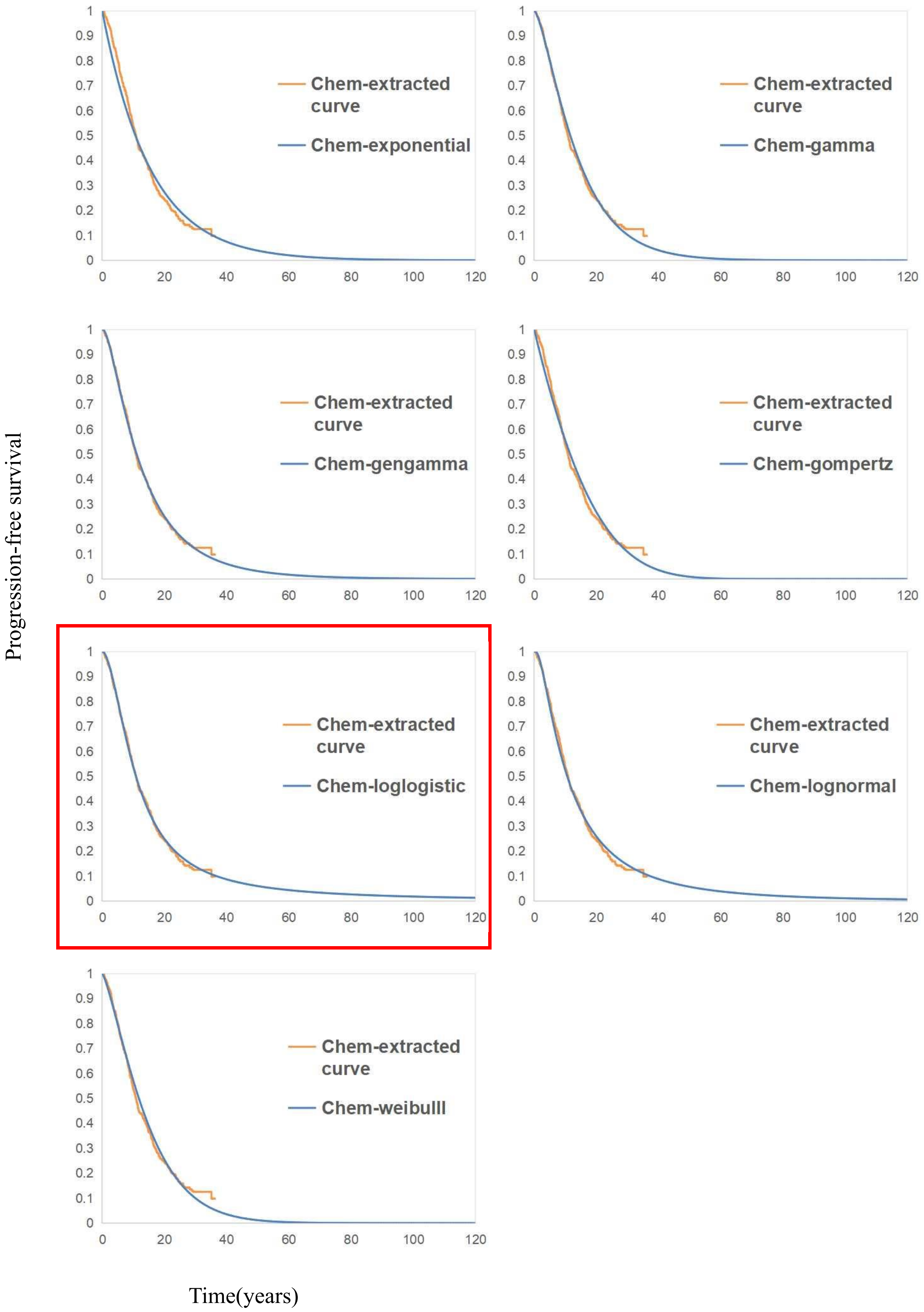

Progression-free survival
Chem-extracted curve
Chem-exponential
Chem-extracted curve
Chem-gamma
Chem-extracted curve
Chem-gengamma
Chem-extracted curve
Chem-gompertz
Chem-extracted curve
Chem-loglogistic
Chem-extracted curve
Chem-lognormal
Chem-extracted curve
Chem-weibulll
Time(years)

Figure S4 Pembrolizumab price simulation

ICER: incremental cost-effectiveness ratio; c: cost; US: United States

China

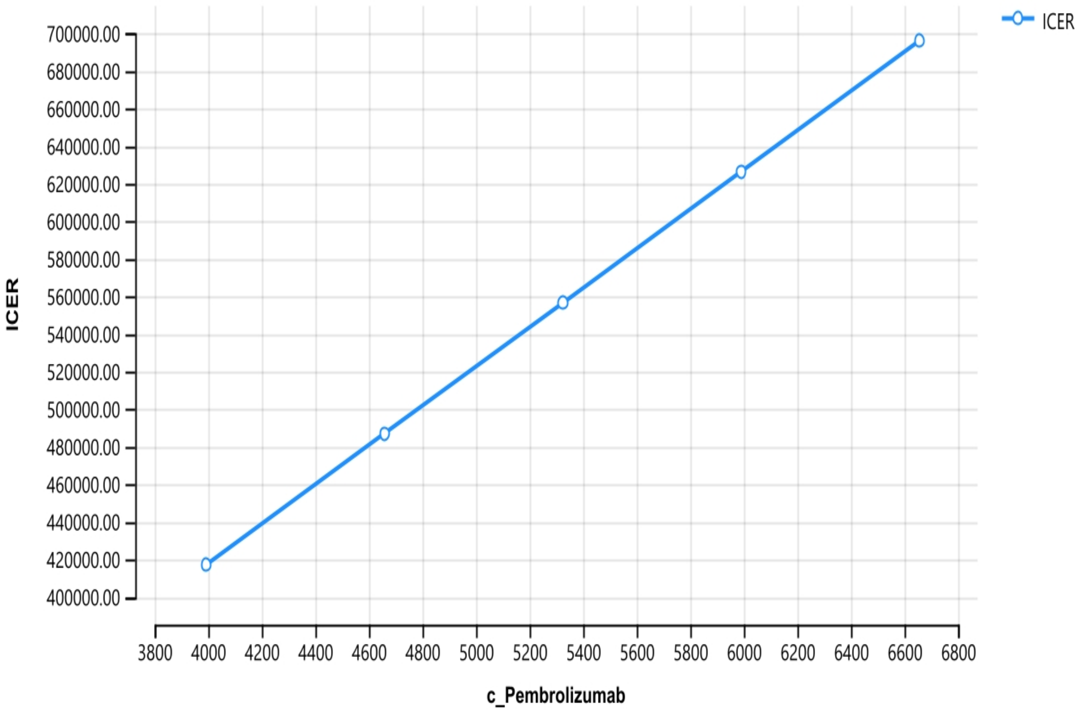

US

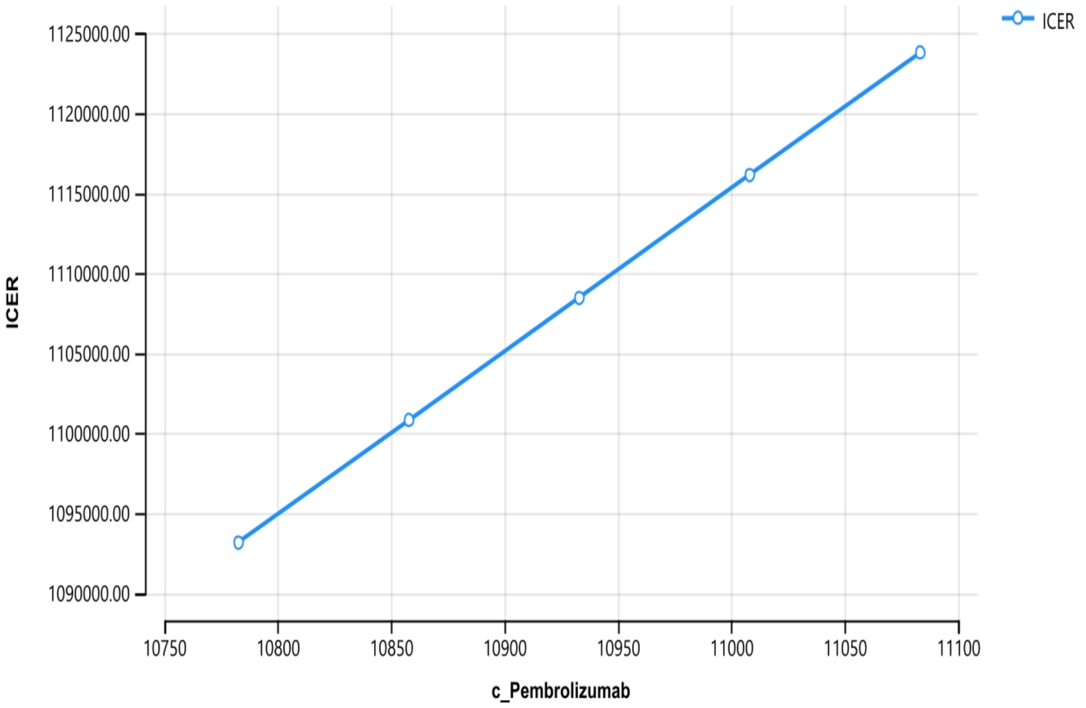

Method

1. Treatment options

The ABC-06 clinical trial isthe first randomised phase 3 clinical trial exploring the role of second-line chemotherapy in advanced biliary tract cancer. It shows a benefit from FOLFOX in terms of overall survival, with a meaningful increase in survival rates at 6 and 12 months. FOLFOX should therefore be considered the standard chemotherapy treatment after progression following cisplatin and gemcitabine(Lamarcaet al., 2021).

REACHIN is the first multicenter, randomized, placebo-controlled, phase II trial to show that regorafenib is active and significantly increases median PFS inpatients with locally advanced/metastatic biliary tract tumors that progress after gemcitabine/platinum-based chemotherapy, whether in second or subsequent lines. Regorafenib could be proposed to patients with severe chemo-derived toxicities (e.g. neurotoxicity of previous platinum therapy or hematologic toxicities)or to those preferring oral therapy(Demolset al., 2020).

A randomised, phase II study evaluated the efficacy and safety of irinotecan-containing regimens in good performance status second-line advanced biliary tract cancer patients. The outcomes from this study showed a clear advantage of XELIRI regimen over irinotecan monotherapy in prolonging PFS, with acceptable toxicity(Zhenget al., 2018).

Pembrolizumab monotherapy provided durable antitumor activity in two separate studies(the KEYNOTE-028 trial, phase 1b; the KEYNOTE-158 trial, phase 2), regardless of PD-L1 expression, and manageable toxicity in patients with advanced biliary tract tumors who had no other options for standard therapy. No unexpected safety signals were observed.Pembrolizumab monotherapy continues as an immunotherapy regimen(Piha-Paulet al., 2020). The above treatment options can be concluded as follow:

Treatment option	Usage
mFOLFOX(max 12 cycles)	Oxaliplatin: 85 mg/m², ivgtt, Q2W<br>L-folinic acid: 175 mg, ivgtt, Q2W (folinic acid: 350 mg, ivgtt, Q2W)<br>Fluorouracil: 400 mg/m², iv and 2400 mg/m², ivgtt 46h, Q2W
Regorafenib	160 mg, qd, d1-21, Q4W
XELIRI	Irinotecan:180 mg/m², d1, Q2W<br>Capecitabine: 1000 mg/m², bid, d1-10, Q2W
Pembrolizumab	200mg, ivgtt, Q3W

2. Supplementary information of key exclusion criteria from KEYNOTE-966

1) Has had previous systemic therapy for advanced (metastatic) or unresectable (locally advanced) biliary tract cancer (intra-or extra hepatic cholangiocarcinoma or gallbladder cancer), with the exception of neoadjuvant/adjuvant therapy which is allowed. Neoadjuvant/adjuvant therapy should have been completed at least 6 months prior to diagnosis of advanced and/or unresectable disease, and participants should not have received gemcitabine and/or cisplatin in the neoadjuvant/adjuvant setting. Participants who received prior neoadjuvant/adjuvant therapy with R2 postoperative pathology of the oncologic resection are excluded.
2) Has ampullary cancer.
3) Has small cell cancer, neuroendocrine tumors, lymphoma, sarcoma, mixed tumor histology and/or mucinous cystic neoplasms.
4) Has an active autoimmune disease that has required systemic treatment in the past 2 years (ie, with use of disease modifying agents, corticosteroids or immunosuppressive drugs). Replacement therapy (eg, thyroxine, insulin, or physiologic corticosteroid replacement therapy for adrenal or pituitary insufficiency, etc.) is not considered a form of systemic treatment and is allowed.

3. Half- Cycle Correction in TreeAge Pro

Real processes occur in continuous time, with transitions and other events occurring throughout an interval of time. However, a Markov process occurs as a discrete sequence of snapshots, which can lead to overcounting Markov rewards. Traditional Markov models accumulate the full cycle's state reward at the beginning of each cycles with transitions understood to occur at the end of each cycle, even though some portion of the cohort will leave the state during the cycle. Epected values will therefore overestimate life expectancy by about half of a cycle (0.5 years in a one-year cycle length model). Consider the simple example of life expectancy in a simple model with health states alive and dead. In each cycle, some portion of the cohort is alive and accumulates 1 life year. In reality, however, deaths will occur halfway through a cycle on average. So, someone that dies during a cycle should lose half of the reward they received at the beginning of the cycle (e.g., -0.5 years of life expectancy in a one-year cycle length model). However, instead of implementing the half-cycle correction as a toll at each transition to death, it is easier to implement it in an absorbing process simply by subtracting a half-reward from the rewards assigned at the beginning of the process, in cycle 0 — i.e., by setting a state's initial reward to one-half of its incremental reward. In a non-absorbing process, in which a significant percentage of the cohort may be alive when the process terminates, cohort members still alive at the end of the process should be given back the half-cycle "death" correction taken from their initial reward at the beginning of the process. This is done by adding on a half-reward after termination in the final reward for all alive states (it does not hurt to always include the initial and final components at every state).

4. The method of calculating the transfer probability of PD status

According to the OS curve, the probability pSTS fro m survival to survival and the probability PSTD from survival to death can be obtained, and the probability pSTS contains three parts, that is, the probability pPTP from progression to progression and the probability pFTF from progression-free to progression-free, so it is necessary to correct, separate the pPTP, and then further obtain the probability pPTD of transition from progression to death by 1-pPTP. The model is calibrated from the second period (the value of the first cycle does not affect the results), and the specific correction formula is as follows:

$$pPTP = \frac{(nPFS + nPD) \times pSTS - nPFS(pFTF + pFTD)}{nPD}$$

Reference

Lamarca A, Palmer D, Wasan H S, Ross P J, Ma Y T, Arora A, et al.Valle J W. (2021). Second-line FOLFOX chemotherapy versus active symptom control for advanced biliary tract cancer (ABC-06): a phase 3, open-label, randomised, controlled trial. *The Lancet Oncology*. 22: 690 - 701. doi.org/10.1016/S1470-2045(21)00027-9.

Demols A, Borbath I, Eynde M V d, Houbiers G, Peeters M, Maréchal R l, et al.Laethem J L V. Regorafenib after failure of gemcitabine and platinum-based chemotherapy for locally advanced/metastatic biliary tumors: REACHIN, a randomized, double-blind, phase II trial. 2020.https://

Zheng Y, Tu X, Zhao P, Jiang W, Liu L, Tong Z, et al.Wang W. (2018). A randomised phase II study of second-line XELIRI regimen versus irinotecan monotherapy in advanced biliary tract cancer patients progressed on gemcitabine and cisplatin. *British Journal of Cancer*. 119: 291 - 5. doi.org/10.1038/s41416-018-0138-2.

Piha-Paul S A, Oh D Y, Ueno M, Malka D, Chung H C, Nagrial A, et al.Doi T. (2020). Efficacy and safety of pembrolizumab for the treatment of advanced biliary cancer: Results from the KEYNOTE ‐ 158 and KEYNOTE ‐ 028 studies. *International Journal of Cancer*. 147: 2190 - 8. doi.org/10.1002/ijc.33013.
